# Association of a Care Coordination Model With Health Care Costs and Utilization: The Johns Hopkins Community Health Partnership (J-CHiP)

**DOI:** 10.1001/jamanetworkopen.2018.4273

**Published:** 2018-11-02

**Authors:** Scott A. Berkowitz, Shriram Parashuram, Kathy Rowan, Lindsay Andon, Eric B. Bass, Michele Bellantoni, Daniel J. Brotman, Amy Deutschendorf, Linda Dunbar, Samuel C. Durso, Anita Everett, Katherine D. Giuriceo, Lindsay Hebert, Debra Hickman, Douglas E. Hough, Eric E. Howell, Xuan Huang, Diane Lepley, Curtis Leung, Yanyan Lu, Constantine G. Lyketsos, Shannon M. E. Murphy, Tracy Novak, Leon Purnell, Carol Sylvester, Albert W. Wu, Ray Zollinger, Kevin Koenig, Roy Ahn, Paul B. Rothman, Patricia M. C. Brown

**Affiliations:** 1 Department of Medicine, Johns Hopkins University School of Medicine, Baltimore, Maryland; 2 NORC at the University of Chicago, Bethesda, Maryland; 3 Johns Hopkins HealthCare, Glen Burnie, Maryland; 4 Johns Hopkins Health System, Baltimore, Maryland; 5 Substance Abuse Mental Health Services Administration, Department of Health and Human Services, Washington, DC; 6 Centers for Medicare & Medicaid Services, Baltimore, Maryland; 7 Sisters Together and Reaching, Baltimore, Maryland; 8 Department of Health Policy and Management, Johns Hopkins Bloomberg School of Public Health, Baltimore, Maryland; 9 Department of Psychiatry, Johns Hopkins University School of Medicine, Baltimore, Maryland; 10 Men and Families Center, Baltimore, Maryland; 11 Johns Hopkins Community Physicians, Baltimore, Maryland

## Abstract

**Importance:**

The Johns Hopkins Community Health Partnership was created to improve care coordination across the continuum in East Baltimore, Maryland.

**Objective:**

To determine whether the Johns Hopkins Community Health Partnership (J-CHiP) was associated with improved outcomes and lower spending.

**Design, Setting, and Participants:**

Nonrandomized acute care intervention (ACI) and community intervention (CI) Medicare and Medicaid participants were analyzed in a quality improvement study using difference-in-differences designs with propensity score–weighted and matched comparison groups. The study spanned 2012 to 2016 and took place in acute care hospitals, primary care clinics, skilled nursing facilities, and community-based organizations. The ACI analysis compared outcomes of participants in Medicare and Medicaid during their 90-day postacute episode with those of a propensity score–weighted preintervention group at Johns Hopkins Community Health Partnership hospitals and a concurrent comparison group drawn from similar Maryland hospitals. The CI analysis compared changes in outcomes of Medicare and Medicaid participants with those of a propensity score–matched comparison group of local residents.

**Interventions:**

The ACI bundle aimed to improve transition planning following discharge. The CI included enhanced care coordination and integrated behavioral support from local primary care sites in collaboration with community-based organizations.

**Main Outcomes and Measures:**

Utilization measures of hospital admissions, 30-day readmissions, and emergency department visits; quality of care measures of potentially avoidable hospitalizations, practitioner follow-up visits; and total cost of care (TCOC) for Medicare and Medicaid participants.

**Results:**

The CI group had 2154 Medicare beneficiaries (1320 [61.3%] female; mean age, 69.3 years) and 2532 Medicaid beneficiaries (1483 [67.3%] female; mean age, 55.1 years). For the CI group’s Medicaid participants, aggregate TCOC reduction was $24.4 million, and reductions of hospitalizations, emergency department visits, 30-day readmissions, and avoidable hospitalizations were 33, 51, 36, and 7 per 1000 beneficiaries, respectively. The ACI group had 26 144 beneficiary-episodes for Medicare (13 726 [52.5%] female patients; mean patient age, 68.4 years) and 13 921 beneficiary-episodes for Medicaid (7392 [53.1%] female patients; mean patient age, 52.2 years). For the ACI group’s Medicare participants, there was a significant reduction in aggregate TCOC of $29.2 million with increases in 90-day hospitalizations and 30-day readmissions of 11 and 14 per 1000 beneficiary-episodes, respectively, and reduction in practitioner follow-up visits of 41 and 29 per 1000 beneficiary-episodes for 7-day and 30-day visits, respectively. For the ACI group’s Medicaid participants, there was a significant reduction in aggregate TCOC of $59.8 million and the 90-day emergency department visit rate decreased by 133 per 1000 episodes, but hospitalizations increased by 49 per 1000 episodes and practitioner follow-up visits decreased by 70 and 182 per 1000 episodes for 7-day and 30-day visits, respectively. In total, the CI and ACI were associated with $113.3 million in cost savings.

**Conclusions and Relevance:**

A care coordination model consisting of complementary bundled interventions in an urban academic environment was associated with lower spending and improved health outcomes.

## Key Points

**Question:**

Is the Johns Hopkins Community Health Partnership, a broad care coordination program inclusive of acute care and community interventions, associated with improved health outcomes?

**Findings:**

This quality improvement study found that the community intervention was associated with a statistically significant reduction in admissions, readmissions, and emergency department visits for Medicaid, but the utilization results were mixed for the acute care intervention. In terms of cost of care, there were statistically significant cost savings totaling $113.3 million.

**Meaning:**

A care coordination model in an urban academic center environment can be associated with improved outcomes, including substantial cost reduction.

## Teaser

This quality improvement study compares the outcomes and costs of Medicare and Medicaid participants who took part in an acute care intervention or a community intervention in Baltimore, Maryland, vs a comparison group drawn from similar Maryland hospitals.

## Introduction

The nearly 200 000 residents of East Baltimore, where life expectancy can be 20 years shorter than in more affluent communities nearby, face multiple challenges to their health and well-being.^1^ Many of these residents receive care at Johns Hopkins facilities, and there are challenges in coordinating care for patients, especially during transitions, related to both medical complexity and underlying social factors. We also know that effective care coordination can lead to improved health outcomes, especially for those of greatest need with chronic conditions.^2,3,4,5,6^ The Johns Hopkins Community Health Partnership (J-CHiP) is a care coordination initiative spanning the care continuum. It has 2 principal program components: (1) a bundle of interventions deployed in 2 acute care East Baltimore hospitals (Johns Hopkins Hospital [JHH] and Johns Hopkins Bayview Medical Center [JHBMC]), with additional focus on those discharged to local skilled nursing facilities (SNFs) and (2) a care management model embedded in ambulatory primary care sites located in the community. The Johns Hopkins Community Health Partnership was catalyzed by a Health Care Innovation Award (HCIA) from the Center for Medicare & Medicaid Innovation, a component of the Centers for Medicare & Medicaid Services, an agency of the US Department of Health & Human Services. Launched in July 2012, external funding for the acute care component (acute care intervention [ACI]) ended in June 2015, while funding for the community component (community intervention [CI]) ended in June 2016.^7^ It is estimated that more than 80 000 participants received services through the award. Previous articles, including a case study, have described the interventions.^7,8^ This article is a follow-up to a report on outcomes as assessed by NORC (formerly the National Opinion Research Center) at the University of Chicago, the independent evaluator for the J-CHiP HCIA award.^9,10^

The ACI, an evidence-based bundle of services, focused on improving the coordination of care for all patients and included (1) early screen for discharge planning to predict service needs following discharge; (2) daily multidisciplinary unit-based rounds to review goals and priorities of care; (3) innovative patient education using tablet-based modules; (4) enhanced medication management, including the option of medications in hand at the time of discharge; (5) telephone follow-up after discharge by nurses staffing a patient access line; and (6) skilled home care, remote patient monitoring, and/or a skilled nurse transition guide for high-risk patients.^11^ Most of these services were deployed across 35 adult inpatient units at either JHH or JHBMC. An additional intervention comprising discharge and disease-based protocols was offered to a subset of patients discharged to 5 local SNFs.

The CI, a care coordination model with integrated behavioral health care, was embedded at 8 ambulatory sites. The intervention used risk prediction models to identify and target Medicare and Medicaid (M/M) patients at greatest risk for future hospitalization. In the original rollout of the intervention, called *J-CHiP Classic,* multidisciplinary teams providing care coordination and community-based health workers (CHWs) were deployed to the neighboring community, working in close partnership with patients’ primary care practitioners. These CHWs focused on addressing patients’ barriers to care, often meeting patients at appointments and in their homes. Health behavioral specialists embedded in the care teams intervened around substance use or other psychiatric diagnoses.^12^ About 1 year into the program, the Tumaini (Swahili for *hope*) for health component was added to the intervention. This component was implemented by 2 community-based organizations, Sisters Together and Reaching^13^ and the Men and Families Center.^14^ Both organizations became key partners in delivering a complementary component of the CI. Sisters Together and Reaching hired and trained an additional nurse case manager and a team of CHWs to supplement those already trained by Johns Hopkins HealthCare, the managed care arm of Johns Hopkins Medicine. The Men and Families Center trained and deployed neighborhood-based support navigators, akin to block captains, in a single census tract to provide outreach and support services to all residents regardless of their insurance status.

For recipients of the ACI or CI, we hypothesized that improved care coordination with team-based deployment would reduce avoidable health care utilization following discharge and in other circumstances, including hospital admissions, readmissions, and emergency department (ED) visits, and would subsequently lead to a reduction in total cost of care from the perspective of the Centers for Medicare & Medicaid Services.

## Methods

### Source of Data and Study Population

Difference-in-differences (DID) study designs were used to study the association between the intervention and health care utilization in the ACI and CI groups. For the ACI, the preintervention period was for claims from January 1, 2011, to June 30, 2012, and the ramp-up period when the intervention was beginning to be implemented was from July 1, 2012, through March 31, 2013; the postintervention period began on April 1, 2013, and M/M claims data through June 30, 2015, were used for the analysis. For the CI, the preintervention period was the 2-year span (8 quarters) before the beneficiary enrolled in the intervention. Medicare claims data for the CI group were available through June 30, 2016, and we used a 90-day claims runoff date of March 31, 2016, for the analysis. Medicaid claims data for the CI group were available through March 31, 2016, with a 90-day claims runoff date of December 31, 2015. Evaluation efforts with respect to the J-CHiP effort were approved by the NORC institutional review board as well as by the Johns Hopkins University institutional review board. A waiver of consent was also granted by the Johns Hopkins institutional review board. This study adhered to the Standards for Quality Improvement Reporting Excellence (SQUIRE) 2.0 reporting guideline.

#### Acute Care Intervention

The acute care analysis compared the associated outcomes of M/M program participants during their 90-day postacute episode with those of a propensity score–weighted preintervention group at J-CHiP hospitals and with a comparison group drawn from similar hospitals in Maryland (Table 1). The Johns Hopkins Community Health Partnership provided an enrollment file of the acute care participants at JHH and JHBMC who were hospitalized in particular inpatient units, which were linked to fee-for-service M/M enrollment and claims data to obtain their demographic and health characteristics and analyze their utilization and cost outcomes. A pool of episodes from these same hospitals during the preintervention period served as the pretreatment group. For the comparison group, a pool of M/M fee-for-service episodes discharged from 3 similar hospitals in geographic proximity to JHH and JHBMC during the preimplementation and postimplementation periods were used. Comparison hospitals were chosen based on case mix and patient demographic characteristics and were all located in Maryland to account for the unique all-payer hospital payment model (eAppendix 1 in the Supplement). We expected that the global budgeting would have similar effects on all Maryland hospitals, but note that being an HCIA awardee, there may be unobserved hospital- and practitioner-level selection effects that are also correlated with outcomes.

**Table 1.** Acute Care Intervention Descriptive Characteristics of Study Sample^a^

Characteristic	Preintervention				Postintervention			
	Medicare		Medicaid		Medicare		Medicaid	
	J-CHiP	Comparison	J-CHiP	Comparison	J-CHiP	Comparison	J-CHiP	Comparison
Beneficiary-episodes, No.	16 316	47 135	11 210	5858	26 144	42 594	13 921	4574
Female, No. (%)	8599 (52.7)	24 510 (52.0)	6143 (54.8)	3251 (55.5)	13 726 (52.5)	22 532 (52.9)	7392 (53.1)	2392 (52.3)
Age, mean, y	67.9	67.5	49.3	52.4	68.4	67.9	52.2	53.0
Race/ethnicity, No. (%)								
White	10 410 (63.8)	30 637 (65.0)	4058 (36.2)	2355 (40.2)	16 994 (65.0)	27 771 (65.2)	5025 (36.1)	1866 (40.8)
Black	5401 (33.1)	15 036 (31.9)	5527 (49.3)	2788 (47.6)	8314 (31.8)	13 502 (31.7)	7406 (53.2)	2191 (47.9)
Other	506 (3.1)	1461 (3.1)	1625 (14.5)	715 (12.2)	810 (3.1)	1320 (3.1)	1503 (10.8)	517 (11.3)
Coverage reason, No. (%)								
Age	9104 (55.8)	26 631 (56.5)	1446 (12.9)	803 (13.7)	14 693 (56.2)	24 193 (56.8)	1142 (8.2)	567 (12.4)
Disability	6559 (40.2)	18 713 (39.7)	9316 (83.1)	4792 (81.8)	10 431 (39.9)	16 739 (39.3)	12 404 (89.1)	3833 (83.8)
Other^b^	652 (4.0)	1791 (3.8)	448 (4.0)	264 (4.5)	1020 (3.9)	1661 (3.9)	376 (2.7)	444 (9.7)
Dual eligibility, No. (%)	5743 (35.2)	16 167 (34.3)	5538 (49.4)	3204 (54.7)	8957 (34.3)	14 439 (33.9)	6334 (45.5)	2324 (50.8)
Health risk score^c^								
Hierarchical Condition Category score, mean (SD)	3.0 (2.1)	3.2 (2.2)	NA	NA	3.3 (2.1)	3.2 (2.2)	NA	NA
Very high Resource Utilization Band score, No. (%)	NA	NA	6636 (59.2)	2982 (50.9)	NA	NA	9870 (70.9)	2433 (53.2)
Utilization and cost in year prior to acute beneficiary-episode, mean (SD)								
Total cost per beneficiary-episode, $	59 931 (90 868)	57 751 (95 082)	38 222 (104 241)	22 040 (59 374)	57 933 (102 299)	56 792 (111 527)	37 150 (80 929)	22 178 (50 057)
Hospitalizations per 1000 beneficiary-episodes	2346.0 (4698.1)	2209.0 (5044.2)	1282.2 (3162.4)	934.1 (2559.1)	2139.3 (4830.9)	1803.5 (4165.2)	1589.2 (3042.4)	1086.0 (2652.9)
Emergency department visits per 1000 beneficiary-episodes	2116.9 (7359.1)	2451.4 (10 515.8)	2329.9 (8495.7)	1702.0 (4798.1)	2244.2 (7238.0)	1704.1 (3891.9)	2783.3 (7931.0)	2044.3 (4538.8)

Abbreviations: J-CHiP, Johns Hopkins Community Health Partnership; NA, not applicable.

^a^ Data reflect results after propensity score weighting.

^b^ Indicates end-stage renal disease with or without disability.

^c^ Adjusted Clinical Group Resource Utilization Band score is used for Medicaid beneficiaries and Hierarchical Condition Category score is used for Medicare beneficiaries.

#### Community Intervention

The analysis of the CI compares the changes in outcomes of M/M program participants before and after their enrollment in the intervention with those of a propensity score–matched comparison group (Table 2). We linked an intervention enrollment file of the CI participants to M/M enrollment and claims data in the preintervention and postintervention periods to obtain their demographic and health characteristics and to analyze their utilization and cost outcomes. As described previously,^7^ the J-CHiP team identified eligible Medicare and Medicaid patients aged 18 years or older with at least 1 chronic condition who received care at a designated primary care site. From this population, high-risk patients were identified based on their risk of future hospitalization. For the Medicare population, we screened 27 000 Medicare fee-for-service patients and calculated hospitalization risk using the ACG (adjusted clinical groups) score. For the Medicaid population, we screened approximately 53 000 Priority Partners Managed Care Organization Medicaid patients using a multivariable logistic regression model to augment the ACG hospitalization score with supplemented data (claims, health plan enrollment, health risk assessment, and lab data). For the comparison group, we analyzed claims to identify a pool of Medicare fee-for-service beneficiaries who lived in or near geographic proximity (defined by the 7 zip codes in East Baltimore where the participating ambulatory sites were located) and who had at least 1 evaluation and management visit to a practitioner within the time period of the community intervention to determine a pseudo–enrollment start date. This allowed us to control for contextual factors that could affect both the treatment and comparison groups. From this pool of potential comparison beneficiaries, selected beneficiaries had at least 1 evaluation and management visit to a practitioner during the year prior to the J-CHiP intervention start date.

**Table 2.** Community Intervention Descriptive Characteristics of Study Sample

Characteristic	Medicare		Medicaid	
	J-CHiP	Comparison	J-CHiP	Comparison
Beneficiaries, No.	2154	2154	2532	2184
Female, No. (%)	1320 (61.3)	1352 (62.8)	1483 (67.3)	1831 (67.9)
Age, mean, y	69.3	69.1	55.1	53.6
Race/ethnicity, No. (%)				
White	903 (41.9)	892 (41.4)	772 (30.5)	636 2(9.1)
Black	1213 (56.3)	1223 (56.8)	1643 (64.9)	1426 (65.3)
Other	39 (1.8)	39 (1.8)	149 (5.9)	114 (5.2)
Coverage reason, No. (%)				
Age	1129 (52.4)	1129 (52.4)	182 (7.2)	166 (7.6)
Disability	946 (43.9)	961 (44.6)	1225 (48.4)	1138 (52.1)
Other	80 (3.7)	65 (3.0)	1124 (44.4)	880 (40.3)
Dual eligibility, No. (%)	1027 (47.7)	1109 (51.5)	1053 (41.6)	845 (38.7)
Enrolled in managed care, No. (%)	NA	NA	1377 (54.4)	1282 (58.7)
Health risk score^a^				
Hierarchical Condition Category score, mean (SD)	2.4 (1.7)	2.2 (1.8)	NA	NA
Very high Resource Utilization Band score, No. (%)	NA	NA	1127 (44.5)	961 (44.0)
Utilization and cost in year prior to program enrollment, mean (SD)				
Total cost per beneficiary, $	34 615 (55 555)	34 151 (119 251)	25 874 (49 759)	26 604 (50 573)
Hospitalizations per 1000 beneficiaries	1192 (2120)	1236 (6118)	900 (1963)	889 (1897)
Emergency department visits per 1000 beneficiaries	1867 (6024)	1491 (3832)	2049 (4817)	2366 (6741)

Abbreviations: J-CHiP, Johns Hopkins Community Health Partnership; NA, not applicable.

^a^ Adjusted Clinical Group Resource Utilization Band score is used for Medicaid beneficiaries and Hierarchical Condition Category score is used for Medicare beneficiaries.

### Outcome Measures

We used claims-based measures for all outcomes, as electronic medical record data were not available for the comparison groups. Additionally, because both the ACI and CI did not target participants with particular clinical profiles (eg, those with diabetes or heart disease) but rather were designed to improve care coordination and care management for participants with broader medical and social needs, we used claims-based measures capturing utilization, quality, and cost of care that reflect these goals. Outcome measures were constructed for each quarter of the study period (eAppendix 2 and eTable in the Supplement). Utilization outcomes were coded as binary events, indicating whether or not the beneficiary had an event during the quarter. Total cost of care (to the payer) was constructed for each 90-day beneficiary-episode (ACI), or for each beneficiary quarter (CI). Utilization measures included all-cause hospital admissions, ED visits, and 30-day readmissions. In addition, we expected the ACI to affect timely receipt of practitioner follow-up (7 and 30 days) after hospital discharge. We assessed physician, nurse practitioner, and physician assistant follow-up visits following hospital discharge as a quality of care measure, as evidence has suggested that 7- and 30-day follow-up visits with practitioners may lead to higher-quality outcomes. This follow-up visit measure does not take into consideration phone calls made to patients via the patient access line after discharge. We expected the CI to have an impact on how participants received care coordination through medical and social supports, and therefore we assessed ambulatory care–sensitive conditions visits and potentially avoidable hospitalizations as quality measures. We computed the former for Medicare and the latter for Medicaid participants because the ambulatory care–sensitive conditions hospitalization measure requires the Present on Admission indicator, which was present on Medicare claims but absent on Medicaid claims. All cost estimates were based solely on data from M/M claims and did not include the cost of the intervention.

### Analytic Design

For the ACI, participants were selected based on having an inpatient stay in a preintervention or postintervention period; the unit of analysis was therefore a beneficiary-episode. For the CI group, participants were selected based on being in the community (and enrolled in the program for the treatment group); therefore, the unit of analysis was the beneficiary. Race/ethnicity were determined and cataloged by claims history for both the ACI and CI. A DID approach was used to estimate the associations between the interventions and utilization and cost outcome measures in each quarter and over the entire preintervention and postintervention period (eAppendix 3 and eFigure 1 in the Supplement). As a DID model is valid when there are no differences in the preintervention period trend, we examined unadjusted data to assess parallel trends and included prior-year outcomes in the propensity score models. In this study, we report aggregate cost measures to show the total savings accrued to M/M over the intervention period. The aggregate measures are the net difference, which is calculated by summing the quarterly impacts weighted by the number of episodes or beneficiaries each quarter. We report quarterly utilization and quality outcomes (average number of events per quarter) per 1000 episodes (ACI) or per 1000 beneficiaries (CI). Quarterly utilization and quality estimates are a weighted average for all quarters in the intervention.

### Statistical Analysis

#### Propensity Score Weighting and Matching

For both the ACI and CI, we used propensity score models to minimize differences between the treatment and comparison groups (eAppendix 4, eFigure 2, eFigure 3, eFigure 4, eAppendix 5, eFigure 5, eFigure 6, and eFigure 7 in the Supplement). In the analysis of the ACI, the propensity score was estimated as the probability of a patient being enrolled in the intervention, conditional on the patient’s covariates. Discharges from both groups during the pretreatment and posttreatment comparison groups were assigned a weight based on the likelihood of being in the posttreatment intervention group. Table 1 shows the descriptive characteristics of the ACI populations after propensity score weighting. In the analysis of the CI, propensity score matching was used to find comparison group beneficiaries comparable to treatment beneficiaries. As with the ACI, the model predicted the likelihood of enrolling in the program. The common support (overlap in the distribution of scores) was then examined, and differences in demographic and health measures in the weighted or matched treatment and comparison groups were assessed to ensure sufficient comparability. Table 2 shows the descriptive characteristics of the CI populations after propensity score matching.

#### Statistical Tests Used

The primary statistical tests for the research question on impact were generalized linear regression models with a gamma distribution or 2-part models with the best-fitting distribution for cost measures, and logit link for binary outcomes, with a prespecified confidence interval of 90% (in accordance with Center for Medicare & Medicaid Innovation guidance). *P* values were noted at *P* < .10 (*P* < .10, *P* < .05, *P* < .01) and were 2-sided.

For some cost categories in the acute care Medicare analyses, 2-part models with the best-fitting distributional form were used. Regression adjustment variables included age category, race/ethnicity, sex, prior-year hospitalizations and cost, dual-eligibility indicator, health risk scores, an indicator of end-stage renal disease, and disability. In the ACI, we also included prior-year hospitalizations and cost and discharge disposition.

Using the regression coefficients, we estimated predicted probabilities; all analyses were conducted in Stata statistical software version 14.0 (StataCorp).

## Results

### Acute Care Intervention

For the ACI, there were 26 144 beneficiary-episodes for Medicare (13 726 [52.5%] female patients; mean patient age, 68.4 years) and 13 921 beneficiary-episodes for Medicaid (7392 [53.1%] female patients; mean patient age, 52.2 years). For Medicare, the ACI was associated with a statistically significant reduction in aggregate cost of care of $29.2 million ($1115 per beneficiary-episode) with increases in 90-day hospitalization and 30-day readmission of 11 and 14 per 1000 beneficiary-episodes, respectively, and reduction in practitioner follow-up visits of 41 and 29 per 1000 beneficiary-episodes for 7-day and 30-day visits, respectively. For Medicaid, the statistically significant reduction in aggregate cost of care was $59.8 million ($4295 per beneficiary-episode), and 90 day ED visit rates were reduced by 133 per 1000 beneficiary-episodes while the hospitalization rate was increased by 49 per 1000 beneficiary-episodes and the practitioner follow-up visits were reduced by 70 and 182 per 1000 beneficiary-episodes for 7-day and 30-day visits, respectively (Table 3). Savings for the Medicaid population were associated with relative reductions in outpatient care and acute care inpatient costs. For the Medicare population, reductions in total cost of care were largely associated with relative reductions in SNF expenses, although there were other contributors as well.

**Table 3.** Medicaid and Medicare Outcomes Associated With the Acute Care Intervention^a^

Outcome	Adjusted Estimate (90% CI)	
	Medicaid (n = 13 921)	Medicare (n = 26 144)
**Utilization**		
Per 1000 beneficiary-episodes		
90-d Hospitalizations	49 (14 to 84)^b^	11 (0 to 22)^c^
90-d Emergency department visits	−133 (−160 to −106)^d^	−10 (−21 to 1)
30-d Readmissions	2 (−29 to 33)	14 (4 to 24)^b^
**Quality**		
Practitioner follow-up visits per 1000 beneficiary-episodes		
7-d	−70 (−92 to −48)^d^	−41 (−51 to −31)^d^
30-d	−182 (−210 to −154)^d^	−29 (−40 to −18)^d^
**Cost, $**		
Aggregate cost^a^	−59 790 132 (−88 987 187 to −30 593 077)^d^	−29 153 336 (–58 468 168 to 0)^c^
Total cost per beneficiary-episode	−4295 (−6392 to −2198) ^d^	−1115 (−2236 to 0)^c^
**Total Cost of Care Categories, $**^a^^,^^e^		
Acute inpatient cost	−32 584 445 (−56 038 104 to −9 130 786)^b^	−5 102 929 (−31 296 679 to 21 090 821)
Skilled nursing facility cost	Not assessed	−11 625 561 (−16 523 340 to −6 727 782)^d^
Other postacute care cost	Not assessed	341 396 (−963 021 to 1 645 813)
Outpatient cost	−14 850 283 (−22 148 552 to −7 552 014)^d^	1 505 092 (−5 385 537 to 8 395 721)
Hospice cost of care	Not assessed	−1 223 164 (−2 474 366 to 28 038)
Durable medical equipment cost	Not assessed	−972 351 (−2 093 598 to 148 896)
Prescription drug cost	−1 518 889 (−3 946 050 to 908 272)	Not assessed

^a^ The aggregate cost of care estimate is the total DID estimate for all program participants across all quarters of program implementation. Quarterly impact is the average quarterly DID estimate per quarter of program implementation. The Medicare and Medicaid analysis is based on 8 quarters of program participation.

^b^ *P* < .05.

^c^ *P* < .10.

^d^ *P* < .01.

^e^ Observed impact for total cost of care categories is not expected to sum to observed impact for the cost categories owing to other costs not examined because of small sample sizes or other types of costs that were not included in the categories shown.

### Community Intervention

A total of 2154 Medicare beneficiaries (1320 [61.3%] female; mean age, 69.3 years) and 2532 Medicaid beneficiaries (1483 [67.3%] female; mean age, 55.1 years) received the CI. For Medicaid, statistically significant aggregate total cost-of-care reduction associated with the CI was $24.4 million (average savings of $1643 per beneficiary per quarter), and reductions of hospitalizations, ED visits, and 30-day readmissions were 33, 51, and 36 per 1000 beneficiaries, respectively, and there was a reduction of avoidable hospitalizations by 7 per 1000 beneficiaries. There were no statistically significant findings for Medicare (Table 4). In total, the CI and ACI were associated with $113.3 million in cost savings.

**Table 4.** Medicaid and Medicare Outcomes Associated With the Community Intervention^a^

Outcome	Adjusted Estimate (90% CI)	
	Medicaid (n = 2532)	Medicare (n = 2154)
Quality		
Avoidable hospitalizations and ambulatory care sensitive hospitalizations per 1000 beneficiaries	−7 (−11 to −3)^b^	0 (−6 to 6)
Cost, $		
Aggregate cost of care	−24 352 777 (−32 665 570 to −16 039 984)^b^	2 238 184 (−4 309 690 to 8 786 058)
Total cost of care per beneficiary	−1643 (−2204 to −1082)^b^	174 (−334 to 682)
Utilization, per 1000 beneficiaries		
Hospitalizations	−33 (−41 to −25)^b^	−5 (−15 to 5)
Emergency department visits	−51 (−62 to −40)^b^	−2 (−12 to 8)
Readmissions	−36 (−64 to −8)^c^	6 (−22 to 34)

^a^ The aggregate cost of care estimate is the total difference-in-differences estimate for all program participants across all quarters of program implementation. Quarterly impact is the average quarterly difference-in-differences estimate per quarter of program implementation per 1000 beneficiaries. The Medicare analysis used 9 quarters for the readmissions measures and 11 quarters for the other outcome measures. The Medicaid analyses used 9 quarters of program implementation.

^b^ *P* < .01.

^c^ *P* < .05.

## Discussion

The J-CHiP program, consisting of multiple complementary and intersecting bundles of strategies deployed within an urban academic environment, was associated with desired outcomes in many utilization, quality, and costs measures for high-risk M/M beneficiaries in East Baltimore by improving the coordination of services across the health care continuum. This was particularly notable with respect to cost savings, and the results showed a statistically significant total cost of care reduction in the ACI for both M/M populations and in the CI for the Medicaid population. Greater aggregate cost savings and lower utilization were found among the Medicaid population than the Medicare population for both the ACI and CI (of note, results of utilization were examined as counts rather than binary events and conclusions were consistent with the binary models).

With respect to the ACI and the Medicaid population, there was a statistically significant increase in hospitalizations, a reduction in ED visits, and an overall statistically significant reduction in total aggregate costs related to reduced acute inpatient and outpatient costs. This suggests the reduced ED visit rate, as well as possibly less intensive acute care resource use during a hospitalization and/or postacute costs, may have contributed to the overall decreased costs. For example, if a hospitalization was required, it was less expensive, likely because of the increased emphasis on early discharge planning, in particular for those with greatest coordination needs. With respect to the ACI and the Medicare population, although hospital use and readmissions increased, there was an overall statistically significant reduction in total aggregate costs, which was likely driven at least in part by a reduction in SNF costs.

Other measures not used in this evaluation (eg, measures of procedures, tests, physician and other staff costs, and community care) may be useful to consider for future impact assessment. However, because of the nature of the evaluation, which required assessment of 107 awardees in total, evaluators used a focused and consistent set of measures; more specific tailoring of outcomes was not within the study’s scope. Finally, it is notable that even with the focus on services following discharge, the results show reductions in practitioner follow-up visits. Because transitional and follow-up care provided by transition guides or care coordinators after discharge was an important intervention component and would not be considered a follow-up practitioner visit, those patients who did not then attend their scheduled follow-up appointment may have done so either because they could not arrange travel or thought it was not needed. The follow-up visits measure reflects completed, and not scheduled, visits.

As mentioned, the cost analyses show that the greatest cost reduction for the Medicare population receiving the ACI was associated with a reduction in SNF utilization. Although related, this was distinct from our additional DID analyses presented in our report that focused on the patients discharged from JHH and JHBMC to 5 partner SNFs near JHH and JHBMC in comparison with those discharged to other SNFs in the same market area.^9^ While we did not find relative savings or lower utilization in this subgroup analysis,^9^ we do observe herein significantly lower SNF costs for those receiving the overall ACI for the Medicare population.

The CI showed a statistically significant reduction in costs, admissions, and ED visits for the Medicaid population, but not for the Medicare population. While there was a trend toward cost reduction for the Medicare population in the final year, this changed in the final quarter, netting a nonsignificant increase in overall costs. The disparate outcomes between M/M patients may reflect the CI focus on patients’ social determinants of health—such as whether the patient can obtain medications, food, or housing—described previously^7^ as a greater need among the Medicaid population. In this context, it is worth considering previously reported findings of subgroup DID analyses of the CI that showed similar outcomes for both the J-CHiP Classic and Tumaini models as well as for patients who differed in their frequency of receiving care coordination services (receiving program staff contact each enrollment quarter vs otherwise).^9^

In addition to our analyses of claims-based outcomes, our evaluation also assessed quality of care using survey data for both clinicians and CHWs.^9^ The Johns Hopkins Community Health Partnership fielded a modified Consumer Assessment of Healthcare Providers and Systems survey for J-CHiP participants in the CI (but who may also have been in the ACI). While these data on patient experience were not collected from the comparison beneficiaries and cannot be attributed solely to J-CHiP, the data provide insight on the process of care delivery. The high survey scores indicate that beneficiaries experienced high levels of quality of care. Respondents reported that their health care practitioner explained things clearly (99%), listened carefully (95%), showed respect (99%), provided easy-to-understand instructions (98%), knew their medical history (95%), and spent enough time with them (98%). Likewise, respondents reported good communication with CHWs, who explained things in a way that was easy to understand (95%), listened carefully (91%), and showed respect for what patients had to say (94%). About 78% of respondents noted that in the previous 12 months they had a discussion with someone in the practitioner’s office about specific goals for their health. Overall, J-CHiP community respondents were extremely satisfied with their practitioners. On a scale of 0 to 10, with 10 being the best, J-CHiP patients rated their health care practitioner an average of 8.9 and rated their trust of their CHW an average of 9.1. About 92% of respondents said they would recommend the CHW to their family and friends.^9^

### Limitations

There were several limitations to this study. Although the ACI was designed to be all-payer, the evaluation focused only on the M/M populations due to data availability. For the CI, enrollment for the comparison group was based on the patient having an evaluation and management visit on the claim; as a result, while both groups have similar baseline utilization and costs, the comparison group, by definition, was as likely, if not more likely, to get care at the time of enrollment as J-CHiP’s participants. It is unclear how selection of the comparison group based on realized ambulatory care may bias the results. In addition, Maryland hospitals where beneficiaries received care were participating in health care delivery reforms such as the Maryland All-Payer Model, which began in January 2014 (midway through the HCIA award period) and involved hospital global budgeting efforts.^15^ The purposeful selection by NORC of Maryland hospitals for the comparison group should minimize the impact of these state reform activities; however, it is possible that the response of each hospital was different, which could bias the results in ways we cannot measure. We acknowledge that unobserved differences in socioeconomic characteristics between Medicare participants in the CI group and the comparison group may bias results toward the null. Even though comparison beneficiaries typically reside in the same general zip code vicinity as those participating in the CI, they are likely to differ on unobserved socioeconomic characteristics. In contrast, our Medicaid CI findings are unbiased, as both comparison and intervention beneficiaries have similar socioeconomic characteristics by virtue of their eligibility for Medicaid. Also, we do not include the cost of the intervention, as this would require a cost-benefit analysis that is beyond the scope of the evaluation and would have necessitated a better understanding of the full scope of associated benefits as well as of costs associated with in-kind contributions. For example, a cost-benefit analysis would entail a collaborative agreement between the J-CHiP implementation team and Center for Medicare & Medicaid Innovation to monetize the return on investment with respect to both direct benefits (beneficiary health, staff training) and indirect benefits (quality of life, improved employment due to better functioning).

In addition to results reported here, there are additional J-CHiP evaluation efforts.^16^ With respect to the ACI, the analysis herein included a focus on intervention units, while additional, currently unpublished analyses look more broadly at the entire target population. The NORC analysis included comparison discharges from selected hospitals in Maryland, while the additional analysis focuses on intrahospital comparisons with yet-to-be-deployed units at the same hospitals. With respect to the CI, this analysis focused on community participants who were touched by a care manager across the entire program duration, while a recently published analysis studied a broader population, including those who may have only received a CHW intervention.

The J-CHiP award was for $19.9 million and included additional institutional investment in these interventions. Statistically significant cost savings achieved by the Centers for Medicare & Medicaid Services were approximately $113.3 million. The initially projected cost savings estimate for the award from the application in 2011 was $52.6 million.^17^ Overall, this suggests a very favorable outcome in terms of cost savings. Nearly all aspects of the J-CHiP award have subsequently been sustained through either state-based initiatives related to the Maryland All-Payer Model^18^ or other initiatives such as the Johns Hopkins HealthCare Medicaid managed care plan or the JHM accountable care organization.^19^

## Conclusions

In summary, the evaluation of the J-CHiP program suggests that a care coordination model that includes separate but complementary bundles of intervention strategies in an urban academic environment can be associated with dramatic improvements in many key utilization and cost indices. However, it is worth noting that the size of the effect is likely not wholly generalizable, as other efforts to implement such care delivery transformations will reflect investments made by the organizations, baseline health and utilization of patients served, and the communities in which they reside. State and federal efforts should continue to focus on best practices, such as those used by J-CHiP, to achieve improvements in health outcomes.

## Notes

Supplement. **eAppendix 1.** Data Source **eAppendix 2.** Measure Specification **eTable.** Measure Specification **eAppendix 3.** Difference-in-Differences Estimation **eFigure 1. **Difference-in-Differences Analysis Design **eAppendix 4. **Propensity Score Methods **eFigure 2. **Acute Care Intervention Medicare Analysis: Common Support and Covariate Balance for J-CHiP and Comparison Beneficiary-Episodes **eFigure 3. **Acute Care Intervention Medicaid Analysis: Common Support and Covariate Balance for J-CHiP and Comparison Beneficiary-Episodes, Dually Eligible **eFigure 4. **Acute Care Intervention Medicaid Analysis: Common Support and Covariate Balance for J-CHiP and Comparison Beneficiary-Episodes, Medicaid Only **eAppendix 5. **Community Intervention: Propensity Score Methods Approach **eFigure 5. **Community Intervention Medicare Analysis: Common Support and Covariate Balance for J-CHiP and Comparison Participants **eFigure 6. **Community Intervention Medicaid Analysis: Common Support and Covariate Balance for J-CHiP and Comparison Participants, Dually-Eligible **eFigure 7. **Community Intervention Medicaid Analysis: Common Support and Covariate Balance for J-CHiP and Comparison Participants, Medicaid Only
