# Supplementary material for: Association of a Care Coordination Model With Health Care Costs and Utilization: The Johns Hopkins Community Health Partnership (J-CHiP)
Source: JAMA Netw Open. 2018 Nov 2;1(7):e184273. doi: 10.1001/jamanetworkopen.2018.4273 (PMC6324376; doi:10.1001/jamanetworkopen.2018.4273)
Supplement: Supplement. — eAppendix 1. Data Source eAppendix 2. Measure Specification eTable. Measure Specification eAppendix 3. Difference-in-Differences Estimation eFigure 1. Difference-in-Differences Analysis Design eAppendix 4. Propensity Score Methods eFigure 2. Acute Care Intervention Medicare Analysis: Common Support and Covariate Balance for J-CHiP and Comparison Beneficiary-Episodes eFigure 3. Acute Care Intervention Medicaid Analysis: Common Support and Covariate Balance for J-CHiP and Comparison Beneficiary-Episodes, Dually Eligible eFigure 4. Acute Care Intervention Medicaid Analysis: Common Support and Covariate Balance for J-CHiP and Comparison Beneficiary-Episodes, Medicaid Only eAppendix 5. Community Intervention: Propensity Score Methods Approach eFigure 5. Community Intervention Medicare Analysis: Common Support and Covariate Balance for J-CHiP and Comparison Participants eFigure 6. Community Intervention Medicaid Analysis: Common Support and Covariate Balance for J-CHiP and Comparison Participants, Dually-Eligible eFigure 7. Community Intervention Medicaid Analysis: Common Support and Covariate Balance for J-CHiP and Comparison Participants, Medicaid Only [file jamanetwopen-1-e184273-s001.pdf]

Supplementary Online Content

Berkowitz SA, Parashuram S, Rowan K, et al; Johns Hopkins Community Health Partnership (J-CHiP) Team. Association of a care coordination model with health care costs and utilization: the Johns Hopkins Community Health Partnership (J-CHiP). *JAMA Netw Open*. 2018;1(7):e184273. doi:10.1001/jamanetworkopen.2018.4273

eAppendix 1. Data Source

eAppendix 2. Measure Specification

eTable. Measure Specification

eAppendix 3. Difference-in-Differences Estimation

eFigure 1. Difference-in-Differences Analysis Design

eAppendix 4. Propensity Score Methods

eFigure 2. Acute Care Intervention Medicare Analysis: Common Support and Covariate Balance for J-CHiP and Comparison Beneficiary-Episodes

eFigure 3. Acute Care Intervention Medicaid Analysis: Common Support and Covariate Balance for J-CHiP and Comparison Beneficiary-Episodes, Dually Eligible

eFigure 4. Acute Care Intervention Medicaid Analysis: Common Support and Covariate Balance for J-CHiP and Comparison Beneficiary-Episodes, Medicaid Only

eAppendix 5. Community Intervention: Propensity Score Methods Approach

eFigure 5. Community Intervention Medicare Analysis: Common Support and Covariate Balance for J-CHiP and Comparison Participants

eFigure 6. Community Intervention Medicaid Analysis: Common Support and Covariate Balance for J-CHiP and Comparison Participants, Dually-Eligible

eFigure 7. Community Intervention Medicaid Analysis: Common Support and Covariate Balance for J-CHiP and Comparison Participants, Medicaid Only

This supplementary material has been provided by the authors to give readers additional information about their work.

© 2018 Berkowitz SA et al. *JAMA Network Open.*

eAppendix 1: Data Source

As described in the main study, our data sources include the Medicare beneficiary enrollment database, Medicare fee-for-service claims (Part A and Part B) located on the Chronic Condition Warehouse (CCW) Virtual Research Data Center (VRDC) and Maryland Medicaid claims from the Maryland Department of Health and Mental Hygiene, provided to us through the Hilltop Institute. We also used files obtained from the J-CHiP Program administrators that identify participants and their enrollment dates. We linked these files to Medicare and Medicaid claims, and created a beneficiary-episode level analytic file for the acute care intervention and a longitudinal beneficiary level analytic file for the community intervention. Each participant-level unit of analysis (beneficiary -episode or beneficiary) contained the cost, utilization, and quality outcome measures for the study.

For the acute care intervention, comparison hospitals were chosen based on case-mix and patient demographics, and were all located in Maryland because hospitals in this state do not participate in the Inpatient Prospective Payment System (IPPS) program. Since Maryland hospitals directly submit claims to Maryland's Health Services Cost Review Commission, which are then forwarded to CMS, inconsistencies in diagnoses have been reported between preliminary and final claims and should be viewed with this in mind. For the community Intervention, the comparison pool consisted of Medicare and Medicaid data (from the VRDC and Hilltop Institute respectively) for FFS beneficiaries living in or near the same seven zip codes in East Baltimore as the J-CHiP participants.

eAppendix 2: Measure Specification

In the table below we provide definitions for the outcome measures used in the evaluation. For the acute care intervention, these measures are calculated at the beneficiary-episode level, and the table in the main report shows the weighted aggregate (weighted by the quarterly estimates) for all *episodes* during the intervention period. For the community intervention the outcomes are estimated at the beneficiary level, and the aggregate is calculated from the (weighted quarterly estimates) for all *beneficiaries* during the intervention period. We used binary measures of utilization, whether an event occurred in each quarter for a specific beneficiary-episode or beneficiary.

eTable: Measure Specification

Measure	Definition
Total Cost of Care	Total Medicare or Medicaid facility and professional costs. For the acute care intervention, these are all costs reimbursed within 90 days of index hospital discharge. For the community intervention, these are all costs payed per quarter.
90-day Inpatient Cost	Medicare or Medicaid facility and professional costs for inpatient care. For the acute care intervention, these are inpatient costs reimbursed within 90 days of index hospital discharge. For the community intervention, these are costs payed per quarter.
90-day Outpatient Cost	Medicare or Medicaid facility and professional costs for outpatient care. Outpatient care includes hospital outpatient, emergency room, observation stays, and office based care, which may include procedures, tests, labs, imaging, and home-based care. For the acute care intervention, these are outpatient costs reimbursed within 90-days of index hospital discharge. For the community intervention, these are costs payed per quarter.
90-day skilled nursing facility cost per beneficiary-episode*	Medicare facility and professional costs associated with skilled-nursing facility (SNF) care within 90 days of index hospital discharge per beneficiary-episode
90-day Other Post-Acute Care Cost per beneficiary-episode *	Medicare facility and professional costs associated with long-term hospital, inpatient rehabilitation, and home health agency care within 90 days of index hospital discharge per beneficiary-episode
90-day Hospice Cost per beneficiary-episode *	Medicare costs associated with hospice care within 90 days of index hospital discharge per beneficiary-episode

© 2018 Berkowitz SA et al. *JAMA Network Open.*

Measure	Definition
90-day Long-term Care Cost **	Medicaid facility and professional costs associated with institutional and home & community based long-term care within 90-days of index hospital discharge per beneficiary-episode
90-day Prescription Drug Cost **	Medicaid costs associated with prescription drugs within 90 days of index hospital discharge per beneficiary-episode
(90-day) Hospitalizations	Hospitalizations for any condition at any hospital. For the acute care intervention, these are inpatient admissions that occur within 90 days of an index hospitalization discharge. For the community intervention, these are inpatient admissions during the quarter.
(90-day) ED Visits	Emergency department visits or observation stays not resulting in hospitalization, for any condition. For the acute care intervention, these are events that occur within 90 days of an index hospitalization discharge. For the community intervention, these are events during the quarter.
30-day Readmissions	Rehospitalizations for any condition at any hospital. For the acute care intervention, these are readmissions that occur within 90 days of an index hospitalization discharge. For the community intervention, these are readmissions during the quarter.
7-day Practitioner Follow-up Visits+	Hospitalizations where a physician, nurse practitioner, or physician assistant follow up encounter occurred within 7 days of index hospitalization discharge
30-day Practitioner Follow-up Visits+	Hospitalizations where a physician, nurse practitioner, or physician assistant follow up encounter occurred within 30 days of index hospitalization discharge
Avoidable Hospitalizations++	Hospitalizations for any of 16 conditions during the quarter. For more details on the conditions and codes, please see: Segal, Misha, et al. "Medicare-Medicaid eligible beneficiaries and potentially avoidable hospitalizations." Medicare & Medicaid research review 4.1 (2014). Available at: https://www.cms.gov/mmrr/Downloads/MMRR2014_004_01_b01.pdf

*Medicare acute care intervention only; **Medicaid acute care intervention only;
+Acute care intervention only; ++Community intervention only.

eAppendix 3: Difference-in-Differences Estimation

We use difference-in-differences (DID) analyses to assess the interventions' effectiveness. This design allows us to estimate the average treatment effect for the programs while limiting the influence of secular trends (by analyzing differences between two groups over the same period).

eFigure 1 below shows how the DID is calculated. The difference in the average outcome between the intervention and the comparison group *after* implementation of intervention **minus** the difference in average outcome between the intervention and the comparison group *before* implementation of the intervention. For more details on this model, please see Appendix C of the HCIA Complex/High-Risk Patient Targeting, Third Annual Report. [1]

[1] HCIA Complex/High-Risk Patient Targeting: Third Annual Report. February 2017. Available at https://downloads.cms.gov/files/cmmi/hcia-chspt-thirdannualrpt.pdf

© 2018 Berkowitz SA et al. *JAMA Network Open.*

eFigure 1: Difference-in-Differences Analysis Design

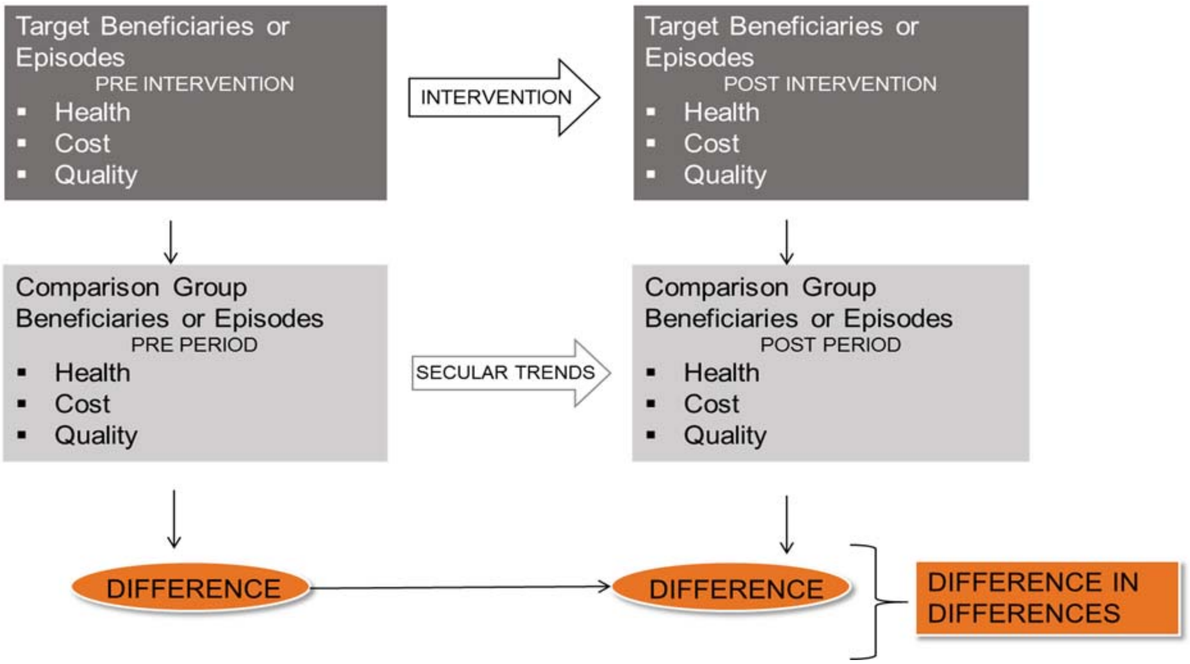

We estimate the double difference by employing mixed logit models for binary outcomes (readmissions, hospitalizations, ED visits, practitioner follow-up visits, and PAH), and generalized estimating equations (GEE) with an appropriate distributional form for cost models. The mixed models are specified using general linearized models with a binomial link and clustered standard errors for the Acute Care intervention, and mixed logit models with population averaged standard errors for the community intervention.

Estimation of Aggregate Impact. The impacts reported for each measure are obtained from DID models, as described above. The *aggregate impacts* are calculated by summing the quarterly impacts, weighted by the number of beneficiary episodes (for the acute care intervention) or beneficiaries (for the community intervention) in the program. The aggregate impacts are presented as the difference in the total number of beneficiary episodes (acute care intervention) or beneficiaries (community intervention) with events (e.g., episodes with 90-day hospitalizations) across the program for utilization and quality of care measures, and the net difference in total cost of care across the program. Net difference is defined as the difference between the treatment and comparison group beneficiaries in the program period, after accounting for differences noted prior to the program period.

eAppendix 4: Propensity Score Methods

Overall

The propensity score models for the Medicare population (Acute Care and Community Interventions) included gender, race, disability status, dual eligibility, Hierarchical Condition Category (HCC) risk score, Medicare Severity-Diagnosis Related Group (MS-DRG) weight and type, cost and ED visits in the year prior to program enrollment. For the Medicaid population (Acute Care and Community Interventions), the propensity score model included age, gender, race/ethnicity, reason for coverage, months of coverage in the final year, and Adjusted Clinical Groups® (ACG) score, and cost and ED visits in the year prior to program enrollment.

Acute Care Intervention: Propensity Score Methods Approach

The propensity score models for the Medicare population (Acute Care and Community Interventions) included gender, race, disability status, dual eligibility, Hierarchical Condition Category (HCC) risk score, Medicare Severity-Diagnosis Related Group (MS-DRG) weight and type, cost and hospitalizations, and ED visits in the year prior to program enrollment. For the Medicaid population (Acute Care and Community Interventions), the propensity score model included age, gender, race/ethnicity, reason for coverage, months of coverage in the final

© 2018 Berkowitz SA et al. *JAMA Network Open.*

year, and Adjusted Clinical Groups® (ACG) score, and cost and ED visits in the year prior to program enrollment. We use propensity score (PS) models to compute ***weights*** for the treatment and comparison beneficiary-episodes to ensure that patients in the two groups are similar with respect to observed covariates. We use relative weighting to maximize the study's power to detect differences by retaining all episodes. Beneficiary-episodes at the awardee site in the pre- and post-intervention period may be systematically different, requiring our PS model to account for four distinct groups: pre-HCIA treatment group, post-HCIA treatment group, pre-HCIA comparison group, and post-HCIA comparison Group. We estimate the PS as the probability of a patient being enrolled in the awardee's program, conditional on the patient's covariates, using multinomial logistic regression. In relative weighting, awardee episodes in the post-intervention period are given a weight of one, while weights for the other three groups are defined as the relative likelihood of being seen by the awardee in the post-intervention period.

We then compute relative weights, and empirically compare the *common support* of propensity scores and the *standardized difference* in the covariates used to compute the propensity score between the J-CHiP and comparison beneficiaries before and after weighting as shown in eFigures 2-4. In the Medicaid analysis (eFigures 3 and 4), we estimated the propensity score models separately for the dually-eligible and Medicaid only populations separately. The eFigures show that after relative weighting, we observe a high level of overlap in the distribution of estimated propensity scores across J-CHiP and comparison pre-intervention and post-intervention beneficiary-episodes.

In the weighted sample, we are able to obtain balance between the four groups on demographic and clinical covariates, comorbidities, and cost and utilization in the year prior to program enrollment.

eFigure 2: Acute Care Intervention Medicare Analysis: Common Support and Covariate Balance for J-CHiP and Comparison Beneficiary-Episodes

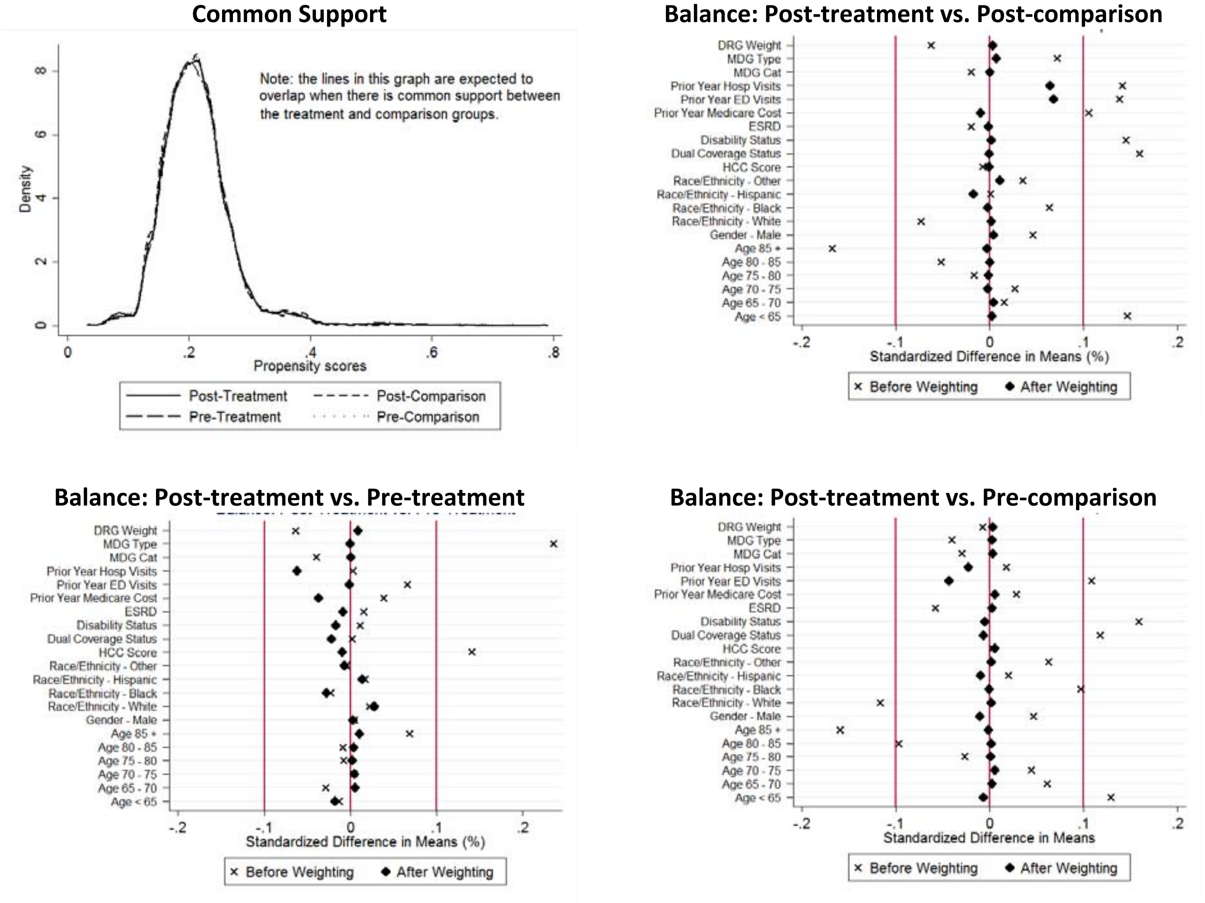

© 2018 Berkowitz SA et al. *JAMA Network Open.*

eFigure 3: Acute Care Intervention Medicaid Analysis: Common Support and Covariate Balance for J-CHiP and Comparison Beneficiary-Episodes, Dually Eligible

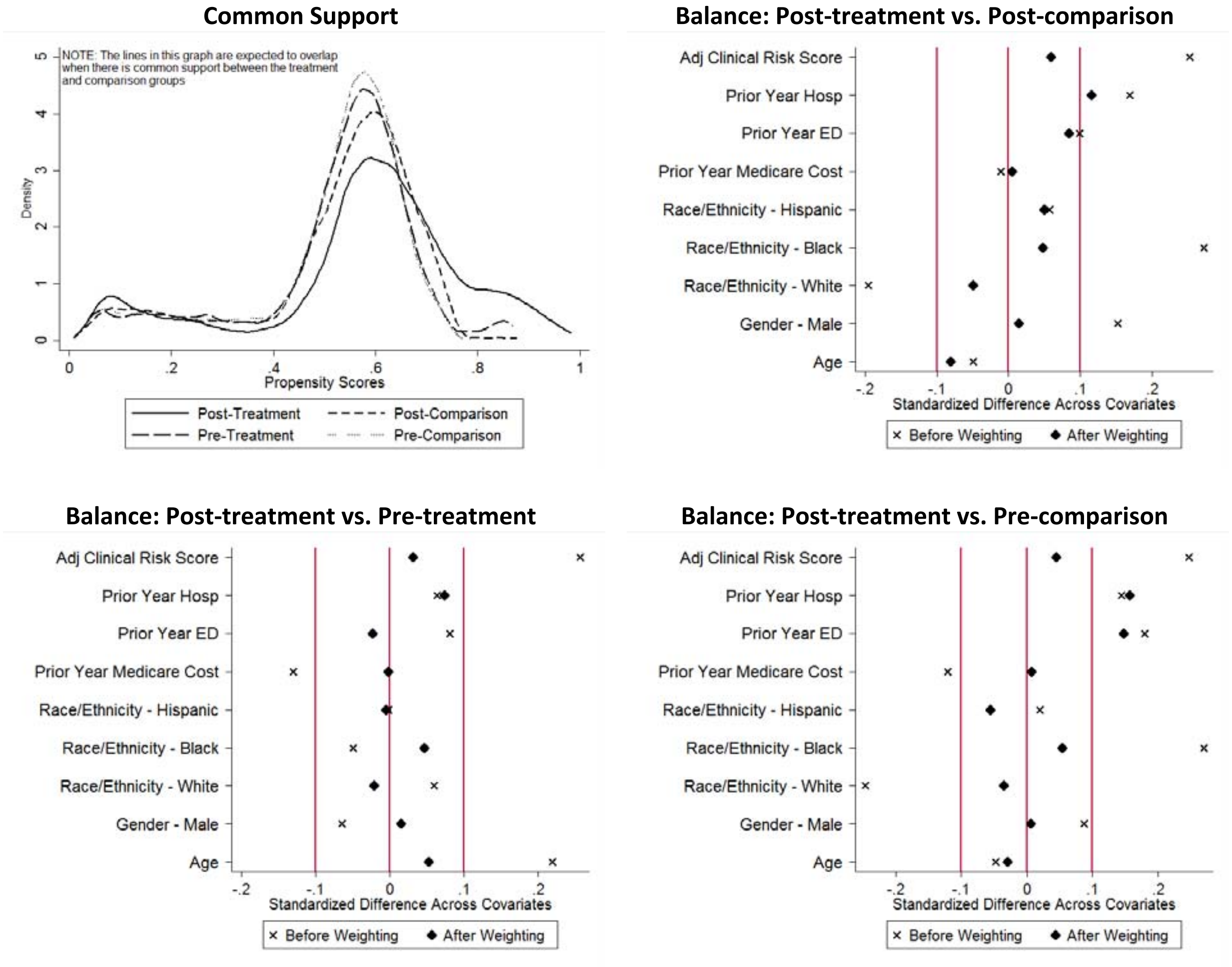

eFigure 4: Acute Care Intervention Medicaid Analysis: Common Support and Covariate Balance for J-CHiP and Comparison Beneficiary-Episodes, Medicaid Only

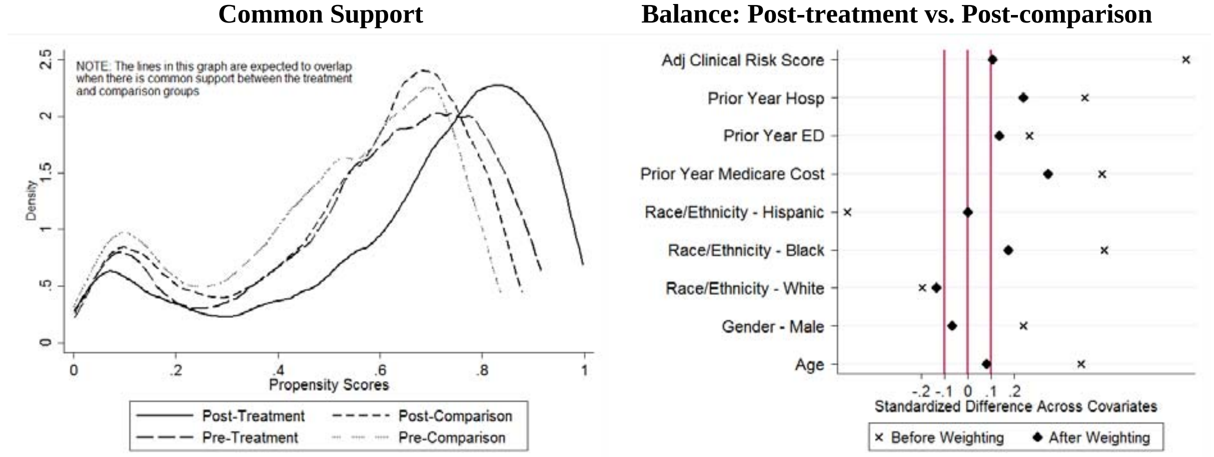

© 2018 Berkowitz SA et al. *JAMA Network Open.*

Balance: Post-treatment vs. Pre-treatment **Balance: Post-treatment vs. Pre-comparison**

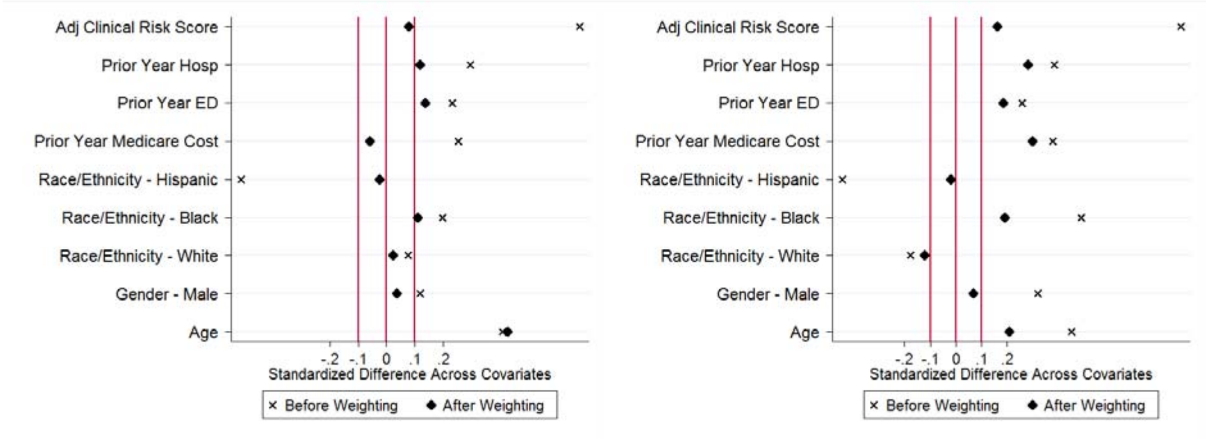

eAppendix 5: Community Intervention: Propensity Score Methods Approach

We use multivariate logistic regression to estimate a PS, or probability of being in the community intervention for community intervention beneficiaries. As mentioned in the report, the model includes a measure of beneficiary demographic and health characteristics, and prior year utilization. After estimating a score, use matching with replacement (Medicaid) or without replacement (Medicare) to find beneficiaries who are “closest” with respect to their propensity score.

As with the relative weighting approach, eFigures 5-7 present common support and covariate balance across the J-CHiP Community intervention treatment and comparison group beneficiaries in the community intervention. After matching, we observe a high level of overlap in the distribution of estimated propensity scores across treatment and comparison groups. In the matched sample, we are able to obtain balance on demographic characteristics, comorbidities, and prior-year utilization. Overall, the chart indicates that propensity score matching greatly improved the comparability of the treatment and comparison groups.

eFigure 5: Community Intervention Medicare Analysis: Common Support and Covariate Balance for J-CHiP and Comparison Participants

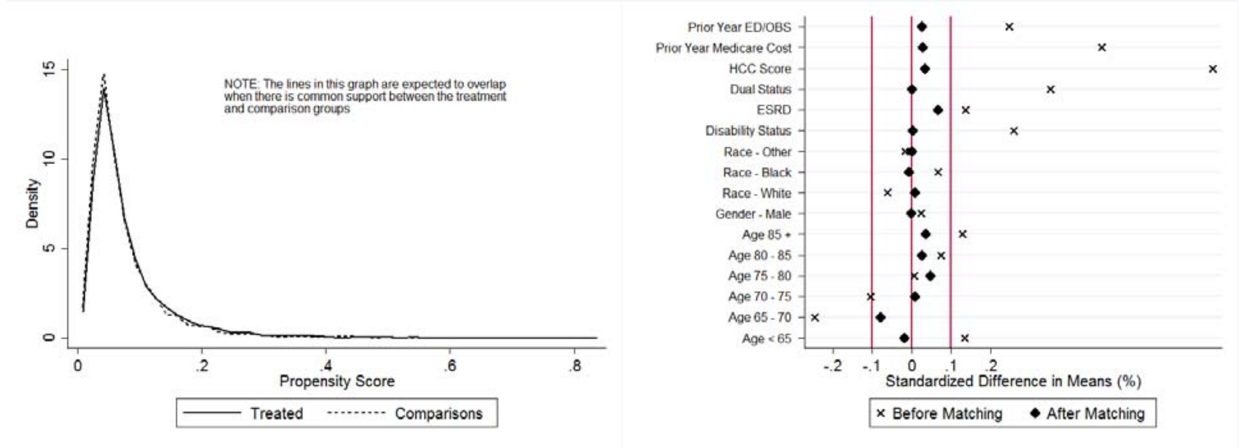

© 2018 Berkowitz SA et al. *JAMA Network Open.*

eFigure 6: Community Intervention Medicaid Analysis: Common Support and Covariate Balance for J-CHiP and Comparison Participants, Dually-Eligible

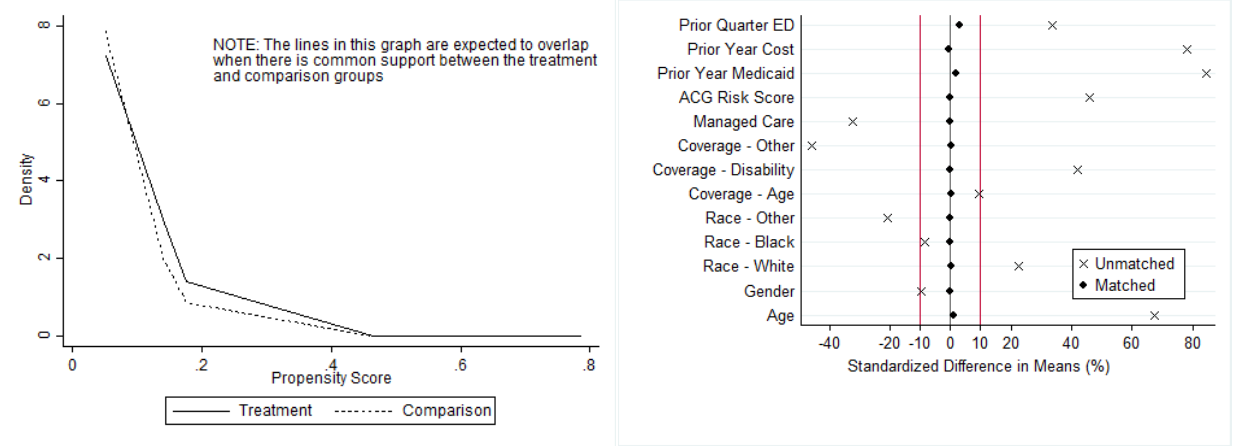

eFigure 7: Community Intervention Medicaid Analysis: Common Support and Covariate Balance for J-CHiP and Comparison Participants, Medicaid Only

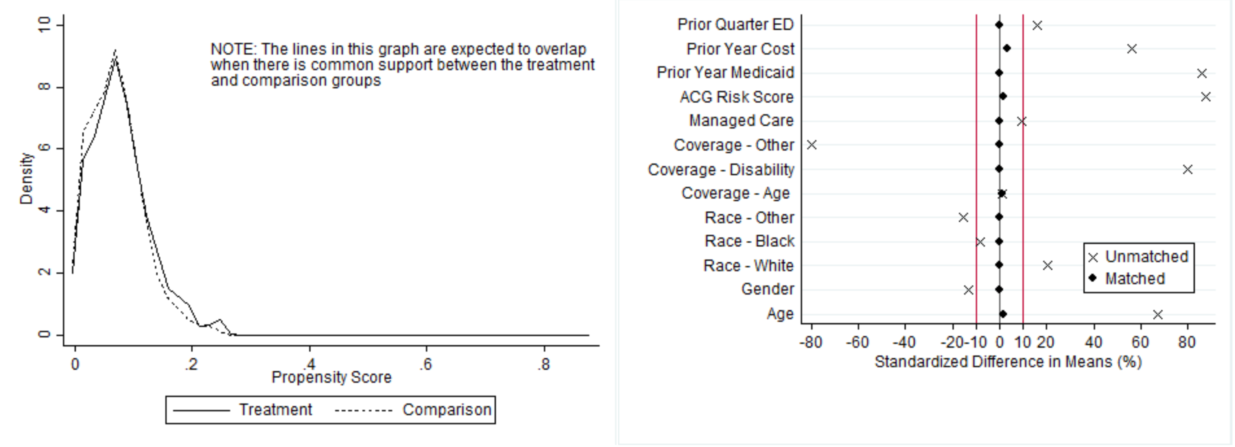

© 2018 Berkowitz SA et al. *JAMA Network Open.*
